# Emerging role of lipid droplets in *Aedes aegypti* immune response against bacteria and Dengue virus

**DOI:** 10.1038/srep19928

**Published:** 2016-02-18

**Authors:** Ana Beatriz Ferreira Barletta, Liliane Rosa Alves, Maria Clara L. Nascimento Silva, Shuzhen Sim, George Dimopoulos, Sally Liechocki, Clarissa M. Maya-Monteiro, Marcos H. Ferreira Sorgine

**Affiliations:** 1 Laboratório de Bioquímica de Artrópodes Hematófagos, Instituto de Bioquímica Médica Leopoldo De Meis, Programa de Biologia Molecular e Biotecnologia, Universidade Federal do Rio de Janeiro, Rio de Janeiro, Brasil; 2 Instituto Nacional de Ciência e Tecnologia em Entomologia Molecular (INCT-EM), Brasil; 3 Instituto Nacional de Câncer, Hospital do Câncer II, Rio de Janeiro, RJ, Brasil; 4 W. Harry Feinstone Department of Molecular Microbiology and Immunology, Bloomberg School of Public Health, Johns Hopkins University, Baltimore, Maryland, USA; 5 Laboratory of Immunopharmacology, Instituto Oswaldo Cruz, Fundação Oswaldo Cruz, Rio de Janeiro, RJ, Brasil

## Abstract

In mammals, lipid droplets (LDs) are ubiquitous organelles that modulate immune and inflammatory responses through the production of lipid mediators. In insects, it is unknown whether LDs play any role during the development of immune responses. We show that *Aedes aegypti* Aag2 cells – an immune responsive cell lineage – accumulates LDs when challenged with *Enterobacter cloacae*, Sindbis, and Dengue viruses. Microarray analysis of Aag2 challenged with *E.cloacae* or infected with Dengue virus revealed high transcripts levels of genes associated with lipid storage and LDs biogenesis, correlating with the increased LDs numbers in those conditions. Similarly, in mosquitoes, LDs accumulate in midgut cells in response to *Serratia marcescens* and Sindbis virus or when the native microbiota proliferates, following a blood meal. Also, constitutive activation of Toll and IMD pathways by knocking-down their respective negative modulators (Cactus and Caspar) increases LDs numbers in the midgut. Our results show for the first time an infection-induced LDs accumulation in response to both bacterial and viral infections in *Ae. Aegypti,* and we propose a role for LDs in mosquito immunity. These findings open new venues for further studies in insect immune responses associated with lipid metabolism.

Lipid droplets (LDs) are multifunctional structures present in most organisms, from bacteria to eukaryotes. These structures are particularly abundant in lipid storing cells, such as mammalian adipocytes and insect fat body cells, in which they were first identified. LDs are mainly composed of a fatty acid monolayer and structural proteins, such as Perilipin 1, Perilipin 2 and 3^1^,^2^. These proteins are involved in LDs biogenesis and degradation, and are exclusively located in LDs, regardless the cell type^3^,^4^,^5^,^6^,^7^. Originally it was believed that the sole function of LDs was the maintenance of lipid homeostasis^8^,^9^. More recently, it has been shown that LDs are also present in mammal immune cells, such as neutrophils and macrophages, where they play important roles in inflammatory or infectious processes, increasing in number upon different types of immune challenges and thereby serving as reliable markers of immune cell activation^10^,^11^,^12^.

In macrophages, the activation of Toll-like receptors (TLR) induces the production of LDs, which concentrate cholesterol esters and triacylglycerol, leading to the formation of foam cells in infected tissues^13^. Also, during viral infections, the activation of TLR induces the recruitment of signaling molecules to LD surface inducing the production of interferons (IFNs), the major mediators of the antiviral response. The inhibition of LDs synthesis prevents adequate IFN production by preventing the formation of signaling transduction platforms^14^. In insects, LDs have not been studied concerning immune-related processes and are considered exclusively as lipid storage organelles^15^,^16^.

Here, using *Ae. aegypti* as a model, we show for the first time that LDs accumulate in response to immune challenges with bacteria and viruses, such as Dengue virus. Using microarray experiments, we confirmed that genes associated with LD biosynthesis and lipid homeostasis are regulated following challenges with Gram-negative bacteria and Dengue. Also, we demonstrate that blood meal induction of native microbiota proliferation triggers LDs biogenesis in the mosquito midgut epithelium. Finally, we show that the constitutive activation of Toll and IMD pathways increase LD content in the mosquito midguts, suggesting that in insects, as in mammals, LDs are directly linked to the activation of immune signaling pathways.

## Results

### Lipid droplets are present in Aag2 cells and are positively modulated by stimulation with Gram-negative Bacteria and Virus

**Figure 1:**
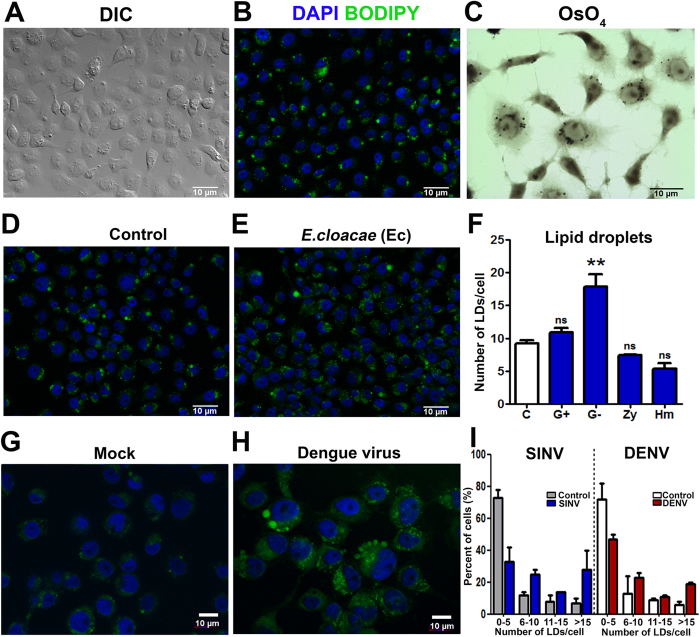
Lipid droplets numbers are modulated in response to Gram-negative bacteria and virus infection in Aag2 cells. (**A**) Differential interference contrast (DIC) or (**B**) BODIPY 493/503 staining for LDs visualization (green) of Aag2 resting cells. Nuclei were stained with DAPI (blue). (**C**) Resting cells were stained with osmium tetroxide (OsO_4_) and LDs can be visualized as black dots in the cytoplasm. (**D**) Resting Aag2 cells or (**E**) cells incubated for 24 hours with heat-killed gram negative bacteria *Enterobacter cloacae*, were stained with BODIPY 493/503 and DAPI. (**F**) Resting Aag2 cells (C - Control) or cells incubated for 24 hours with heat killed gram positive (G + , Micrococcus luteus), gram negative (G−, Enterobacter cloacae) bacteria, zymosan (Zy) or 20 μM heme (Hm) were stained with osmium tetroxide and the number of LDs per cell following different types of stimuli were counted on an optical microscope and represented in the graph. **(G,H)** Aag2 cells mock infected (**G**) or infected with Dengue 2 virus (**H**) were stained with BODIPY 493/503 and DAPI for LDs visualization. (**I**) Aag2 cells were infected with Dengue 2 for 7 days or Sindbis virus for 4 days and stained with osmium tetroxide for LD counting. Figure represents the percentage distribution of the numbers of LDs per cell. In (**F**) for statistical analyses, we used ANOVA followed by Dunnett’s multiple comparison test.

We confirmed large numbers (average 9 LD/cell) of LDs in the cytoplasm of resting Aag2 cells, through staining with BODIPY 493/503 and Osmium tetroxide (OsO_4_), two stains commonly used for LD identification (Fig. 1A–C).

In leukocytes, an increase in the number of cytoplasmic LDs is considered as an indicator of immune activation^11^. We tested whether Aag2 cell LD were influenced by immune stimulation through exposure to heat-killed Gram-negative (*Enterobacter cloacae*) or Gram-positive (*Micrococcus luteus*) bacteria, Zymosan, a polysaccharide from yeast walls, and heme, which represents a non-immunogenic but cell-damaging stimulus. After 24 hours exposure, cells were stained with OsO_4_ or BODIPY 493/503 and the number LDs was determined in each condition (Fig. 1D–F, S1 and S2). We found that heat-killed Gram-positive bacteria did not alter the number of LDs, but the Gram-negative bacteria *Enterobacter cloacae* promoted a significant increase in the number of LDs in the Aag2 cell cytoplasm (Fig. 1D–F and S1 and S2). Exposure to Zymosan and heme negatively modulated the number of LDs in these cells, as previously observed for mammalian macrophages^17^ (Fig. 1F, S1 and S2). These findings were also confirmed by flow cytometry, when cells were submitted to the same treatments, and incubated with the fluorescent probe BODIPY 493/503 (See Supplementary Fig. S1 online).

Since *Aedes aegypti* mosquito is the major vector of yellow fever and dengue viruses, we tested whether these viruses were capable of modulating the number of LDs in Aag2 cells. Aag2 cells were infected with two different arboviruses, the alphavirus Sindbis, and the flavivirus Dengue for 4 and 7 days, respectively, prior to determination of LD numbers using the osmium tetroxide or BODIPY 493/503 stains (Fig. 1G–I). Interestingly, infection of cells with both viruses led to an increase of LDs in the cytoplasm when compared to mock-infected cells (Fig. 1G–I and S3).

### Expression of genes involved in lipid metabolism and LD biogenesis is modulated by challenges with Gram-negative bacteria and Dengue virus

**Figure 2:**
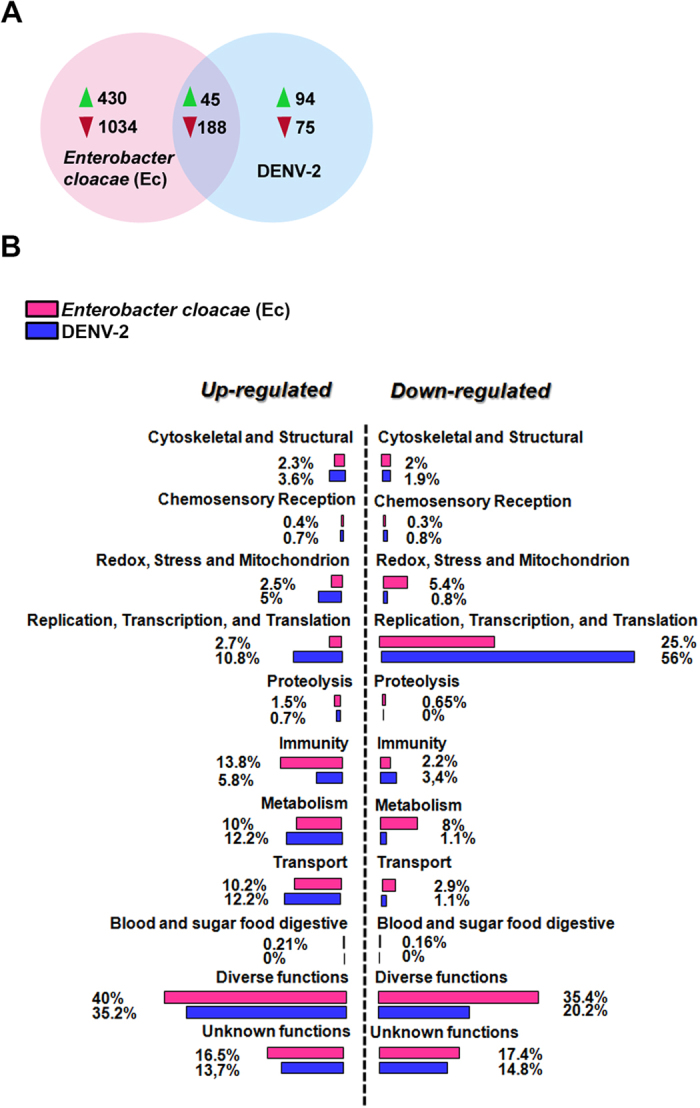
Several *Aedes aegypti* genes are modulated by infections with both gram negative bacteria and virus. Aag2 cells were exposed to either heat-killed *Enterobacter cloacae* (Gram negative, Ec) for 6 hours or to Dengue virus (*Halstead strain*) for 4 days. After these periods total RNA was used in a microarray analysis aiming to identify genes regulated in both conditions. (**A**) Venn diagram showing genes modulated by bacterial infection (pink circle, left) viral infection (blue circle, right) or modulated by both infections (intersection of the circles). The green arrowhead indicates the number of up-regulated genes and the red arrowhead the number of down-regulated genes in each condition. (**B**) Functional classification of the genes modulated by bacterial or viral infection. The graph shows the functional class distribution of modulated genes, obtained using gene ontology software. (http://www.geneontology.org/). See Dataset S1 for complete gene expression changes dataset.

To probe into the molecular mechanism involved in the observed LD increase upon challenges with Gram-negative bacteria or viruses, we used a whole-genome oligonucleotide microarray to compare the transcriptomes of non-challenged Aag2 cells to cells that had been exposed to either heat-killed *Enterobacter cloacae* (Gram-negative) bacteria for 6 hours or to Dengue virus for 4 days. Exposure of the Aag2 cells to *Enterobacter cloacae* significantly regulated 1697 genes (475 genes were induced and 1222 repressed), while Dengue virus infection significantly regulated 432 genes (139 were up-regulated and 263 down-regulated) (Fig. 2 and see Supplementary file Dataset S1 online). Interestingly, the modulation of genes related to lipid metabolism and LD biogenesis went in opposite directions between the two challenges. Most of the proteins involved in lipid metabolism, storage and transport, such as Apolipoprotein D, Fatty Acid Synthase, Annexin X and Malic enzyme, were up-regulated in cells stimulated with the bacterial challenge and suppressed upon Dengue infection. Surprisingly, genes related to LD biosynthesis such as LSD-1 and LSD-2, insect homologs of Perilipin 1 and 2 genes respectively, presented the same pattern. Thirteen genes that were regulated by both challenges were analyzed by qPCR to validate the robustness of the microarray-based transcript abundance assays (see Supplementary Fig. S4 online).

### *In vivo*, LDs are positively modulated by stimulation with Gram-negative Bacteria or Viral infection in the mosquito midgut

**Figure 3:**
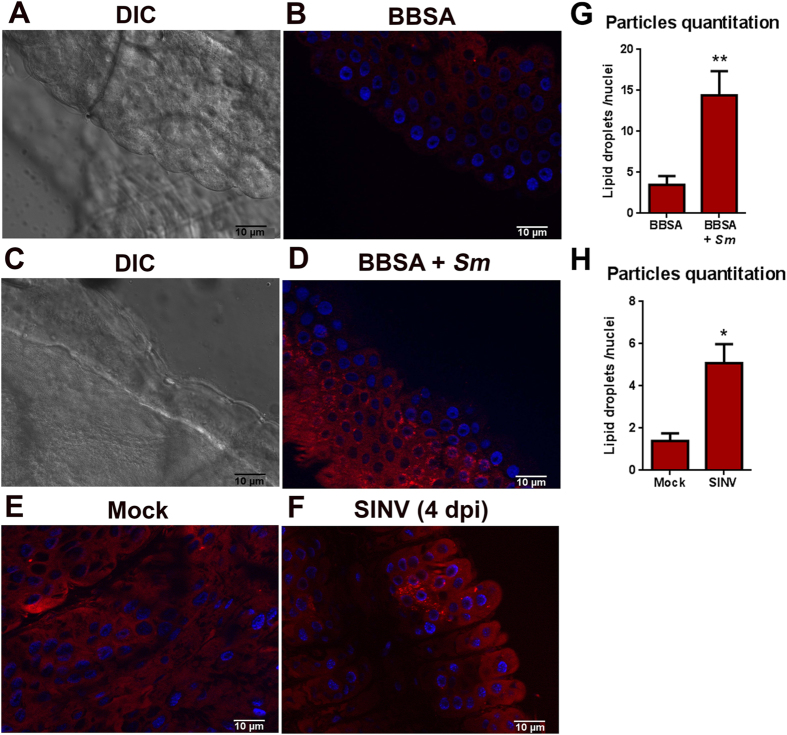
Bacterial and viral infections lead to an increase in the number of LDs in mosquito midgut. *Aedes aegypti* females were fed with a saline solution containing live *Serratia marcescens* and 24 hours after infection, midguts were stained with Nile Red for visualization of LD numbers. (**A**,**C**) Differential interference contrast (DIC); (**B,D**) Nile red stained midguts (red). Alternatively, *Aedes aegypti* females were fed with Sindbis virus and 4 days post infection, midguts were stained with Nile Red. (**E**) Mock infected mosquitoes; (**F**) Sindbis infected mosquitoes. Nuclei were stained with DAPI (blue). (**G**,**H**) Fluorescent particle quantification was performed using Analyze particles tool from ImageJ.

Next, we investigated whether infection-mediated immune challenge of live mosquitoes would also result in LD enrichment as was observed for Aag2 cell line. Blood sucking insects typically acquire blood-borne pathogens through the blood meal or microbes through the nectar meal, and the midgut is the first tissue known to mount a local immune response upon this exposure^18^,^19^. To evaluate if an immune challenge would also lead to an increase of LDs in the mosquito midgut, we fed *Aedes aegypti* females with a saline solution (BBSA) containing the entomopathogenic Gram-negative bacteria *Serratia marcescens.* Twenty-four hours after infection, midguts were dissected, and LD enrichment was estimated through staining with Nile Red (a fluorescent probe that stains neutral lipids, and, consequently, LDs (Fig. 3A–D). As expected, infection with Gram-negative bacteria led to an increase in the number of LDs in the cytoplasm of the midgut cells (Fig. 3A–D). The number of fluorescent particles in each condition was quantified, and midgut cells stimulated with *Serratia marcescens* presented three times more LDs in their cytoplasm than controls (Fig. 3G).

To evaluate the influence of midgut infection with the virus on LD enrichment, *Aedes aegypti* females were infected by feeding on Sindbis virus-supplemented rabbit blood. Enrichment of LDs was then assayed 4 days post infection using the Nile red stain. Accordingly, Sindbis virus-infected females showed a high number of cytoplasmic LDs in their midgut cells, compared to non-infected control mosquitoes that had fed on naive blood (Fig. 3E,F). Unlike the stimulation with Gram-negative bacteria, when all cells throughout the tissue were stimulated, with virus infection, an increased number of LDs were only detected in some groups of cells of this tissue. To evaluate the amplitude of induction, we quantified the number of fluorescent particles for each condition. SINV infected tissues presented an average of 2.5 more LDs than mock infected tissues (Fig. 3H). Thus, immune responsive LD modulation occurs in both adult mosquitoes and Aag2 cells.

### The *Aedes aegypti* midgut microbiota stimulates LD enrichment in midgut cells

**Figure 4:**
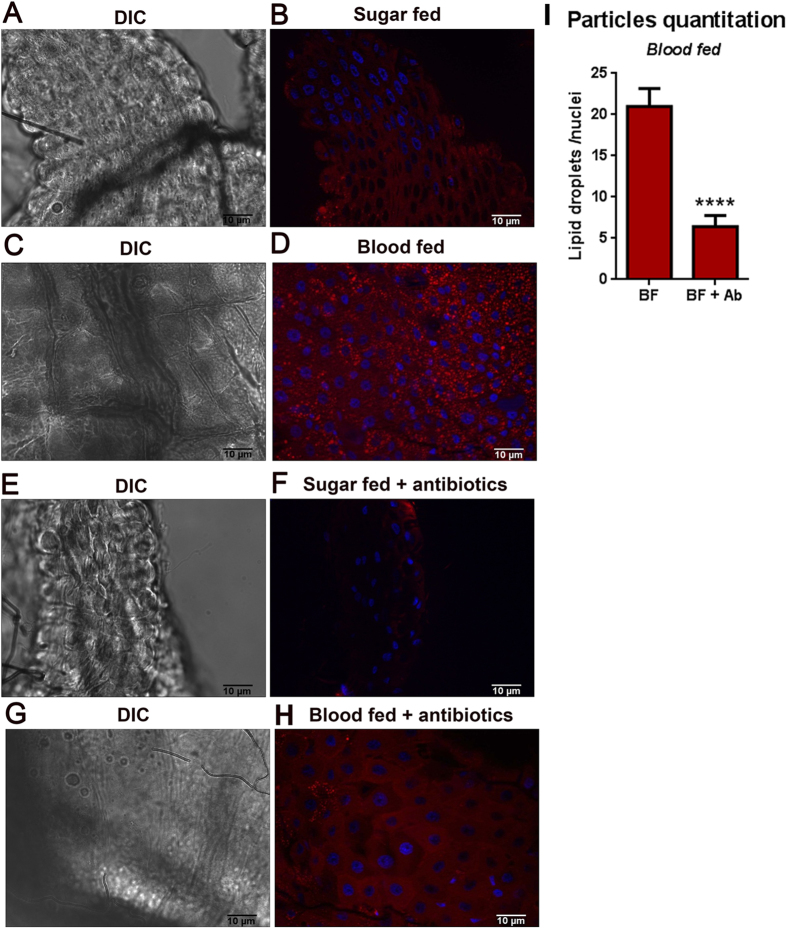
The growth of gut microbiota that follows a blood meal increases the amount of midgut lipid droplets. Mosquitoes were kept feeding on a sugar solution (**A,B**) or blood fed (**C,D**) and 24 hours after feeding, midguts were stained with Nile Red. Alternatively, mosquitoes were fed with antibiotics added to the sugar solution for four days, after which they were kept feeding on the same solution (**E,F**) or blood fed (**G,H**). After additional 24 hours midguts were stained with Nile Red. (**A,C,E,G**) Differential interference contrast (DIC). (**B,D,F,H**). Nile red stained lipid droplets (red). Nuclei were stained with DAPI (blue). (**I**) Fluorescent particle quantification was performed only for blood-fed conditions (with and without antibiotics) using Analyze particles tool from ImageJ.

The *Aedes aegypti* midgut microbiota is composed mainly by gram-negative bacteria^20^,^21^,^22^. After a blood meal, the bacterial load of the midgut can increase by up to one thousand-fold, thereby representing a major immune challenge to the mosquito^20^,^23^. To investigate whether the increase of the midgut native microbiota following a blood meal would also lead to an enrichment of LDs, *Aedes aegypti* female midguts were dissected and stained with Nile Red 24 hours after blood ingestion, revealing a huge increase in the amount of cytoplasmic LDs (Fig. 4B,D). A mosquito cohort that had been maintained on antibiotic-supplemented (penicillin and streptomycin) sugar solution for 4 days prior to blood feeding was used as a control. Treatment of mosquitoes with antibiotics depleted most of the microbiota from females midguts^23^. Even in the absence of a blood meal, antibiotic treatment lead to a significant decrease in the amount of LDs in the midgut cells cytoplasm (Fig. 4B,F). Analysis of the midgut cells of blood-fed females from both cohorts revealed a significantly higher number of cytoplasmic LDs in the non-antibiotics treated mosquitoes compared to the antibiotics treated ones (Fig. 4D,H). The comparison between fluorescent particles of blood-fed mosquitoes previously treated or not with antibiotics revealed that the antibiotic treatment leads to a 4-fold fluorescence decrease in comparison with untreated blood-fed ones (Fig. 4I). Our results indicate that the increase in LDs that follows a blood meal is mainly a response to the growth of the indigenous microbiota that follows the blood meal and not only an attempt to store lipids obtained from the meal.

### LD increase involves fatty acid production and triacylglycerol accumulation

**Figure 5:**
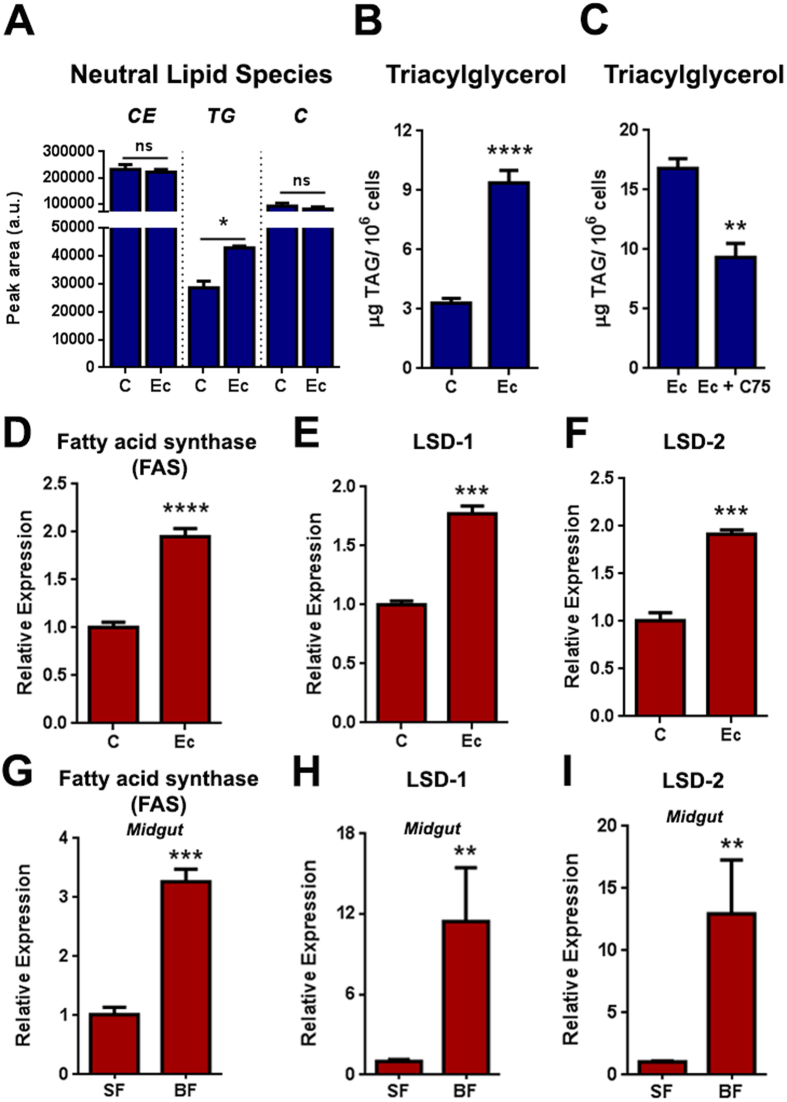
LD enrichment involves fatty acid synthesis and storage in the form of triacylglycerol. Aag2 cells were incubated or not with heat killed Ec and 24 hours later lipids were extracted and either fractioned using a TLC for neutral lipids (**A**) or cells were lysed and subjected to enzymatic determination of triacylglycerol contents (**B**). Alternatively, Aag2 cells were incubated with heat killed Ec in the presence or absence of 10 μg/ml of C75 (a Fatty acid synthase inhibitor). After 2  hours cells were lysed and subjected to enzymatic determination of triacylglycerol contents (**C**). In a different experiment, Aag2 stimulated or not with heat killed Ec (**D–F**) or midguts from mosquitoes fed only on sugar or with blood (**G–I**) had their total RNA extracted and the expression of Fatty acid synthase (FAS) (**D,G**), Lipid storage droplet −1 (LSD-1) (**E,H**) or Lipid storage droplet −2 (LSD-2) (**F,I**) genes was determined by qPCR.

To determine which species of neutral lipids accumulated upon LD increase, we challenged Aag2 cells with heat-killed *E.cloacae* (Ec) and then performed a thin layer chromatography (TLC) for neutral lipids. Densitometry analysis of the TLC revealed that cholesterol and cholesterol ester were the main lipid species present within the cell in both Aag2 control and challenged with bacteria (Fig. 5A and Supplementary Fig. S5 online). Interestingly, an increase in triacylglycerol (TG) amounts can be noticed in heat killed Ec challenged cells (Fig. 5A). Measurement of total TG content by an enzymatic colorimetric assay confirmed this result. In fact, Aag2 cells stimulated with heat killed Ec presented three times more triacylglycerol in comparison to control cells (Fig. 5B), while the fatty acid synthase (FAS) inhibitor, C75, partially prevented the increase of TG in heat killed Ec challenged Aag2 (Fig. 5C). In agreement with these results, we also detected an up-regulation of FAS, as well as, LDs associated structural genes, LSD-1 and LSD-2 in heat killed Ec challenged Aag2 (Fig. 5D).

The same pattern was observed comparing midgut of sugar and blood-fed mosquitoes 24 hours post feeding. FAS expression was three times higher in the midgut of blood-fed mosquitoes (Fig. 5G) and LSD-1 and LSD-2 presented an induction of approximately 10 fold in comparison to sugar-fed mosquitoes (Fig. 5H,I).

### Activation of the Toll and IMD pathways lead to LD enrichment in mosquito midgut cells

**Figure 6:**
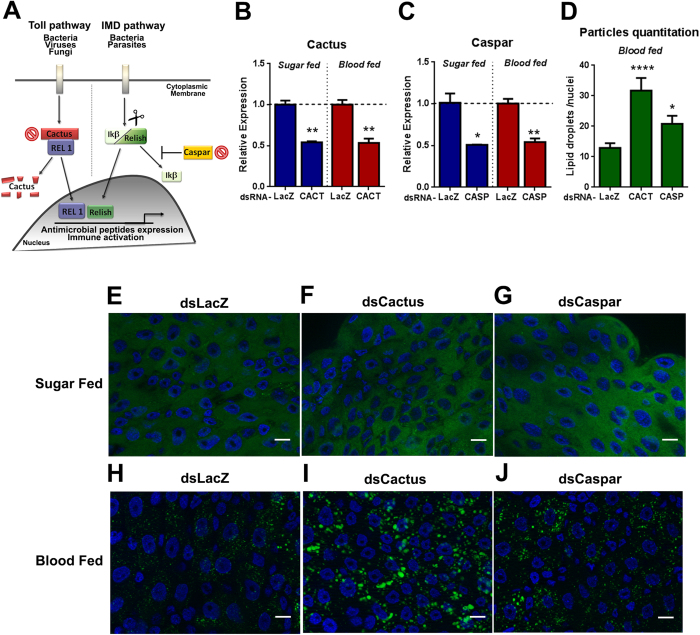
Immune pathways activation induces LD accumulation in mosquito midgut cells after a blood meal. Mosquitoes were injected with double-stranded RNA (dsRNA) for Cactus, Caspar and LacZ (a control dsRNA). 24 hours post injection mosquitoes were either maintained feeding on sugar or fed with blood. After 24 hours, midguts were dissected for RNA extraction or BODIPY 493/503 staining for visualization of LDs. (**A**) Schematic representation of the insect immune pathways, Toll and IMD. Red crosses represent genes that were knocked-down using RNAi, Cactus and Caspar. (**B**) Knock-down efficiency in the mosquito midgut injected with dsCactus (**B**) and dsCaspar (**C**) in both sugar-fed and blood-fed conditions was determined by qPCR. Gene expression is relative to tissues from dsLacZ injected mosquitoes (evidenced by the dashed line). (**E–G**)BODIPY staining of lipid droplets from sugar fed mosquito midguts from insects injected with dsLacZ (**E**), dsCactus (**F**) and dsCaspar (**G**). BODIPY staining of lipid droplets from blood fed mosquito midguts from insects injected with dsLacZ (**H**), dsCactus (**I**) and dsCaspar (**J**). (**D**) Fluorescent particle quantification from images **(H–J)** was performed using Analyze particles tool from ImageJ. Scale bar = 10 μm.

Next, we investigated if LD enrichment in mosquito midgut cells had any relation with the activation of classical invertebrate immune pathways, Toll and IMD. To address this question, we used RNAi technology to knock-down the expression of the negative regulators of Toll and IMD pathways, Cactus and Caspar, respectively. It is known that the knockdown of Cactus and Caspar leads to activation of Toll and IMD pathways^24^ (Fig. 6A). After double-stranded RNA (dsRNA) injection, mosquitoes were kept feeding on sugar or received a blood meal. After 24 hours, midguts were dissected to evaluate knock-down efficiency, which was of approximately 60% in the midgut of both sugar and blood-fed mosquitoes for both genes, when compared with mosquitoes injected with control (dsLacZ) (Fig. 6B,C). Alternatively, 24 hours after blood ingestion, blood and sugar-fed mosquito midguts were stained with BODIPY 493/503. In sugar-fed midguts, no changes were observed in mosquitoes injected with dsRNA for Cactus or Caspar when compared to dsLacZ injected mosquitoes (Fig. 6E–G). Nevertheless, in midguts of blood-fed mosquitoes a significant increase in cytoplasmic LDs content was observed in both Cactus and Caspar dsRNA-injected mosquitoes when compared to dsLacZ injected ones (Fig. 6H–J). This increase was particularly significant in mosquitoes that had the Toll pathway activated through injection of dsCactus RNA (Fig. 6I). We quantified the fluorescence particles and determined that midguts injected with dsCactus had three times more LD content compared to blood-fed dsLacZ injected (Fig. 6D). Midguts from mosquitoes injected with dsCaspar showed a slight but significant increase in comparison to dsLacZ blood-fed tissues (Fig. 6 D,H,J). Together these results show a direct link between classical immune pathways and LD biogenesis in mosquitoes.

## Discussion

Here we show for the first time that, as in mammals, immune competent insect cells and tissues also increase the number of LDs upon different immune stimuli, as a marker of immune activation.

When the *Aedes aegypti* immune-competent Aag2 cells^25^, are stained with different agents that stain neutral lipids (OsO_4_, BODIPY 493/503 or Nile Red)^26^, the presence of LDs becomes evidenced in the cell cytoplasm (Fig. 1A–C). Interestingly, challenging cells with different immune elicitors, such as gram negative bacteria or Dengue and Sindbis viruses, but not with Gram-positive bacteria or zymosan, leads to a significant increase in the number of LDs (Fig. 1D–I, see Supplementary Fig. S1 and S2 online). An increase in cytoplasmic LDs was observed when cells were treated with heat-killed bacteria, implicating the recognition of some PAMP characteristic of Gram-negative bacteria by cell receptors with the induction of this phenomenon, most likely DAP-type peptidoglycan^27^. This is very similar to what can be observed in mammals, where LPS, a major component of Gram-negative bacteria outer membrane, leads to an increase of LDs through activation of TLR4^28^. In mammals, the same pattern is also observed for stimulation with *Mycobacterium Bovis BCG,* through activation of Toll-like receptor 2 (TLR-2)^29^ or viruses such as dengue^30^. Exposure to zymosan, although activating TLR2 is unable to induce an increase of LDs^29^ in mammals, as observed in mosquito cells. This response suggests specificity toward different types of stimuli, also in insects.

Aiming to identify proteins related to the modulation of the amount of LDs that follows immune challenges we exposed Aag2 cells to gram negative bacteria or Dengue virus and performed a microarray analysis. Bacterial infection modulated 1697 genes in the cell line while Dengue virus infection regulated 432 genes, evidencing that the stimulation with bacteria modulated the global gene expression profile in a much more pronounced way than Dengue infection (Fig. 2). This was expected, because virus survival depends on its replication and maintenance into the live cell host and it has been shown that Dengue virus infection inhibits, to a certain extent, *Aedes* immune response^31^. Interestingly 233 genes were regulated in both conditions (45 up-regulated and 188 down-regulated), suggesting that these may be genes involved in a response common for both pathogens, resembling the behavior observed for LDs. Interestingly, when these genes are classified based on their ontological category, it can be noticed that the percentage of genes regulated by both immune challenges involved in metabolic processes potentially related to LD assembly is comparable to the percentage of regulated genes from categories known to be related with infection control, such as immunity and antioxidant response, revealing an important role of lipid metabolism, and probably of LDs, in the response against these pathogens. When the modulated lipid-related genes are analyzed in detail, genes involved in lipid metabolism, storage, and transport and classically related to LDs’ biogenesis and structure can be identified. As an example, Aag2 cells stimulated with Ec presented a pronounced up-regulation of genes such as Perilipin 1(LSD-1) and Perilipin 2 (LSD-2), genes that code for proteins that are exclusively located in LDs and are considered markers of this structure^5^,^6^,^16^,^32^,^33^,^34^,^35^,^36^. Guo *et al.* (2008)^37^ identified several genes related to LD formation and function in Drosophila. Interestingly many genes identified as essential for LDs physiology in this study were also modulated by the immune challenges used in our work, such as COPI and Arf proteins, Fatty acid synthase, phospholipases A and D and GTPase-activating proteins (GAPs) (Fig. 2, see Supplementary Information Fig. S4 and Dataset S1 online).

Leukocytes from human patients with severe dengue present an increased number of LDs^38^. The same phenomena can be observed in Dengue 2 infected BHK cells^30^. We also observed that Aag2 cells infected with both Dengue and Sindbis virus presented a higher number of LDs than control cells (Figs 1G–I and S3). Besides, when we analyzed the number of LDs in the midgut cells of a Sindbis virus-infected mosquito, we could identify several cells with numerous LDs, a feature that was not identified in non-infected mosquitoes (Fig. 3E,F,H). Nevertheless, it is noteworthy that the LD distribution pattern observed in Sindbis infected mosquitoes is different from the one observed in *Serratia* fed ones, where the whole epithelium, and not only some cells, presented numerous LDs (Fig. 3B,D). This is expected and consistent with the dynamics of Sindbis infection because during the first days of infection, the midgut is not homogeneously infected, and only some foci of virus replication can be evidenced in the tissue^39^.

Recently, several groups published data analyzing the composition of *Aedes aegypti* indigenous microbiota, and all of them concluded that it is composed mainly of Gram-negative bacteria^20^,^21^,^22^. For this reason, it was not a surprise that, after a blood-meal, an event that is followed by an increase of midgut bacterial levels of up to 1000 fold, the number of LDs was also greatly increased (Fig. 4A–D). To determine if the observed LD increase was actually due to the immune challenge represented by the growing bacteria and not simply a requirement for the storage of the lipids absorbed from the blood-meal, mosquitoes were treated with an antibiotic mixture to reduce its native midgut microbiota and blood-fed. Even in the absence of a blood meal, just treating the mosquitoes with antibiotics already leads to a very clear reduction in the number of LD present in the tissue (Fig. 4B,F). This effect becomes even more evident after the blood-meal, when numerous LDs can be seen in the cytoplasm of control mosquitoes while very few can be identified in antibiotics treated ones. This points that LD increase is caused by the immune challenge represented by the increased bacterial levels and not by the need to store the lipids absorbed from the blood (Fig. 4D,H,I).

Bacterial infections promote macrophage activation leading to an increase in cytoplasmic LDs. This process is triggered by TLR activation and leads to accumulation of cholesterol esters and triacylglycerol inside the LDs^40^. Aag2 cells present a similar neutral lipid pattern when stimulated with heat killed Ec, accumulating cholesterol ester (CE), cholesterol (C) and triacylglycerol (TG) (Fig. 5A). In mammals, macrophages are able to modulate the amount of each neutral lipid species depending on its physiological condition. For example, both hypoxia and stimulation with oxidized LDL (oxLDL) lead to macrophage LDs accumulation, but the lipid content for each condition is different^41^. Hypoxia induces LDs enriched with triacylglycerol while oxLDL induces accumulation of cholesterol esters^41^. In our model, Aag2 cells challenged with heat killed Ec presented increased in triacylglycerol content but no changes in cholesterol and cholesterol ester content (Fig. 5A,B). Inhibition of the enzyme fatty acid synthase (FAS), with C75, promotes significantly reduction in triacylglycerol amounts showing that *de novo* fatty acid synthesis is requires for triacylglycerol accumulation and is related to LD enrichment observed in response to a bacterial challenge (Fig. 5C). Also, Aag2 cells incubated with heat killed Ec had increased gene expression of FAS, when compared to control cells (Fig. 5D), reinforcing the idea that fatty acid synthesis is important for triacylglycerol storage within the cell. *In vivo*, midguts from blood fed mosquitoes also presented a higher expression of FAS (Fig. 5G), indicating that probably fatty acid synthesis is important for LDs enrichment in the mosquito midgut. LD associated structural genes, LSD-1 and LSD-2, up-regulation in both Aag2 stimulated with heat killed Ec and midgut from blood-fed mosquitoes, showed that, besides lipid synthesis, the synthesis of LD structural proteins is also required for LD enrichment (Fig. 5E,F,H,I).

In mammals, the connection between TLR activation pathway and the modulation of LDs is well established. In dendritic cells, activation of TLR7 leads to the recruitment of molecules from TLR cascade itself to the surface of the LD in the cytoplasm. In this case, LDs function as platforms to assembly signal transduction that leads to interferon production during viral infections^14^. Constitutive activation of the mosquito Toll and IMD pathways led to LD enrichment after blood ingestion in comparison to LacZ control blood-fed mosquitoes (Fig. 6D,H–J). Activation of the Toll pathways leads to a more pronounced increase when compared with Caspar silenced blood-fed midguts (Fig. 6D,I,J). This indicates that, in mosquitoes as in mammals, LD increase in the midgut is linked to the activation of Toll and IMD pathways (Fig. 6D,H–J). Also, TLR activation is directly linked to triacylglycerol retention and accumulation in human and murine macrophages, generating foam cells at sites of infection and inflammation^13^. We hypothesize that after blood ingestion, the microbiota proliferation leads to LD enrichment in midgut cells that is triggered by Toll pathway activation resulting in the production of antimicrobial peptides and immune activation. Previous work showed that in *Aedes aegypti,* constitutive activation of the Toll pathway leads to anti-Dengue effect, reducing virus amounts in the midgut^24^. We believe that this effect could be related to LD enrichment and Toll activation in the midgut. Also, microbiota proliferation is known to antagonize Dengue virus infection in the midgut of *Aedes aegypti*. We could also believe that this antiviral effect is a product of tri-partite interaction between the microbiota proliferation, Toll and IMD activation and LD enrichment leading to the establishment of the immune response.

Altogether, the results presented in this paper show, for the first time that, as observed in mammals, insects also rely on lipid droplets as part of the cellular components required for the production of an effective antibacterial and antiviral immune response. We believe that the description of lipid droplets as important players in virus and bacterial infection responses in the mosquito may reveal new targets for blocking insect infection and further block disease transmission to humans.

## Material and Methods

### Ethics statement

All animal care and experimental protocols were approved by the institutional care and use committee -Comitê para Experimentação e Uso de Animais da Universidade Federal do Rio de Janeiro/CEUA-UFRJ – (Protocol number: CEU-UFRJ # 155/13) and the NIH Guide for the Care and Use of Laboratory Animals (ISBN 0–309-05377-3). All methods were carried out in accordance with the approved relevant guidelines and regulations.

### Mosquitoes and Cell Culture

*Aedes aegypti* (Red Eye strain) were raised in an insectary at the Federal University of Rio de Janeiro, Brazil, under a 12 h light/dark cycle at 28 ^o^ C and 70–80% relative humidity. Larvae were fed dog chow. Adults were maintained in cages and fed a solution of 10% sucrose *ad libitum*. Four- to seven-day-old females were used in the experiments. *Aedes aegypti* Aag-2 cells were maintained at 28 ^o^ C in Schneider´s *Drosophila* medium with L-glutamine (Life Tecnologies, Grand Island, NY) supplemented with 10% Fetal Bovine Serum (FBS) (Cultilab, Campinas, SP).

### Cell Treatments and Viral Infection

For bacterial challenge, cells were incubated with two different heat-killed bacteria as previously described^42^: *Micrococcus luteus*, a Gram positive bacteria, and *Enterobacter cloacae*, a Gram negative bacteria. Aag-2 cells were incubated with 100 bacteria per cell (10^7^ bacteria/well). Cells were also incubated with 0.5 mg/mL Zymosan A (Sigma-Aldrich, St. Louis, MO), as previously described^43^. Cells were also stimulated with 20 μM heme, a cell damaging molecule, prepared in phosphate buffer. For viral infection, cells were infected with Sindbis virus or Dengue virus (DENV) using a MOI (Multiplicity of Infection) of 1, as previously described^44^.

### Mosquito Meals

Mosquitoes were artificially fed with different diets: (1) 10% sucrose (*ad libitum*), (2) rabbit blood, (3) bicarbonate-buffered saline-agarose (BBSA) supplemented with *Serratia marcescens* (Sm). The BBSA solution was composed of glucose (10 mg), 500 mM freshly made bicarbonate buffer pH 7.4 (10 mL), 0.5 mg low melting-point agarose and 100 mM ATP, pH 7.4 (5 mL). The final volume was set to 500 mL with 150 mM NaCl. Feeding was performed using water-jacketed artificial feeders maintained at 37 ^o^ C and sealed with a Parafilm “M” (Sigma-Aldrich, St. Louis, MO) membrane. For depletion of mosquito’s microflora, females were fed with sucrose 10% supplemented with antibiotics, penicillin (100 u/mL) and streptomycin (100 μg/mL) (LGC biotecnologia, Cotia, SP) for 4 days as previously described^23^.

### Mosquito gene knock-down by RNAi

Double-stranded RNA was synthesized using MEGAscript T7 transcription kit (Ambion) from fragments amplified from cDNA of whole mosquitoes using specific primers containing a T7 tail (See supplementary Table S1). Mosquitoes with 3–4 days were injected with the double-stranded RNA and 24 hours post injection were fed with blood. LacZ gene was used as a non-related control and was amplified from a plasmid containing the LacZ fragment cloned.

### Lipid droplet staining and quantification

Aag-2 cells were seeded in 24-well plates and at indicated times, cells were fixed in formaldehyde 3.7% diluted in phosphate saline buffer (PBS). For LD staining with osmium tetroxide (OsO_4_) cell slides were stained following protocols previously described^26^. The total amount of lipid droplets was determined by counting the number of lipid droplets per cell in 50 cells of different fields. Alternatively, cells were incubated with 1 μM BODIPY (493/503 4,4-difluoro 1,3,5,7,8 pentamethyl 4-bora3a,4a-diaza-s-indacene) (Life Technologies, Grand Island, NY). In this case, LD content was evaluated using either fluorescence microscopy (Zeiss AxioObserver, Oberkochen) or flow cytometry (FACSCanto II flow cytometer, Becton/Dickinson Biosciences, Qume Drive San Jose, CA). For midgut lipid droplet staining, tissues were incubated with Nile Red (9-diethylamino-5 H-benzo[alpha]phenoxazine-5-one) (Sigma-Aldrich, St. Louis, MO) at 1:5000 dilution from stock solution. Female midguts were dissected, fixed with 4% paraformaldehyde and then stained with Nile red solution for 10 min. Cells and tissue slides were mounted with VECTASHIELD mounting medium with DAPI (Vector labs, Burlingame, CA). Cells and tissues were observed in a fluorescence microscope Zeiss Axioskop 40 with an Axiocam MRC5 using a Zeiss-15 filter set (excitation BP 546-612; beam splitter FT 580; emission LP 590) for Nile Red and Zeiss-15 and Zeiss-10 (excitation BP 450–490; beam splitter FT 510; emission BP 515–565) for BODIPY 493/503. Differential interference contrast (DIC) images were acquired with a Zeiss AxioObserver microscope. For flow cytometry analysis, cells stained with BODIPY 493/503 were analyzed in a FACS Canto II flow cytometer equipped with FACSDiVa software (BD). Data analysis was performed with the Infinicyt software (Cytognos, Salamanca, Spain).

### Triacylglycerol measurement in Aag2 cells

Aag2 cells were maintained in 25 cm^2^ flasks and incubated or not with heat killed *E. cloacae* in the presence or absence of C75, a fatty acid synthase inhibitor, during 24 hours. After incubation, cells were removed with a cell scraper and centrifuged at 1500 rpm at room temperature for 10 minutes. Media was removed and cell pellet was ressuspended in 100⌌l of sterile milliQ water. Cell lysis was performed through consecutive thawing and freezing using dry ice. Samples were stored at −70 ^o^ C until the day of the assay. The enzymatic assay for triacylglycerol was performed with Laborclin triglicérides GPO trinder (Laborclin produtos para laboratorio ltd.) according to the manufacturer protocol.

### Quantification of fluorescent particles

Fluorescent particles were measured with ImageJ, using Analize Particles tool. After subtracting the background, a specific threshold was set for each experiment. This type of analyses was only performed in induced LD conditions because the threshold of sugar-fed conditions had variable levels in comparison to LD induced conditions. The number of fluorescent particles was normalized by the nuclei numbers for each field.

### Isolation and analysis of neutral lipids by Thin Layer Chromatography (TLC)

Total lipids were extracted from Aag2 cultured cells according to the method Bligh and Dyer^45^ for TLC analysis. The solvent was removed by N_2_ gas, the lipids were dissolved in chloroform/methanol (1:1, v/v) and spotted onto a Silica Gel 60 thin-layer chromatography (TLC) plate. The TLC plate was developed in hexane- diethyl ether-acetic acid (60:40:1, v/v) and neutral lipids were visualized by iodine vapor and 30% sulfuric acid aqueous solution and heat. Neutral lipids were identified according to TLC standards (Cholesteryl Oleate, Cholesteryl and Glycerol trioleate – ICN Biomedicals Inc) and semiquantified by densitometry in NIH ImageJ software.

### Microarray Gene Expression Analysis

Aag-2 cells were seeded to a confluence of 80% in 12-well plates and treated in quadruplicate with the following:

(a) DENV *Halstead strain* (propagated in *Aedes albopictus* C6/36 cell line) at an MOI of 1. (b) Supernatant of C6/36 cells without DENV (mock-infected). (c) Heat-killed Gram negative bacteria *Enterobacter cloacae*. (d) Schneider´s *Drosophila* medium.

After incubation at 28 ^o^ C, for 96 hours of conditions (a) and (b) and for 6 hours of conditions (c) and (d), infected and control cells were lysed by the addition of 600 mL of Buffer RLT (Qiagen) and homogenized for 30 s with a rotor-stator homogenizer. RNA was then extracted with the Qiagen RNeasy Mini Kit. Two micrograms of total RNA were used for synthesis of Cy3- and Cy5-labeled cRNA probes and hybridizations were carried out on an Agilent-based microarray platform. Hybridization intensities were determined with an Axon GenePix 4200AL scanner, and images were analyzed with Gene Pix software. Expression data were processed and analyzed as previously described^24^,^46^. Self–self-hybridizations have been used to determine the cutoff value for the significance of gene regulation on these types of microarrays to 0.78 in log2 scale, which corresponds to 1.71-fold regulation^47^. Numeric microarray gene expression data are presented in Supplementary Dataset S1.

### RNA Extraction, cDNA synthesis and Quantitative PCR

Total RNA from cells in all conditions was extracted using the TRIZOL reagent (Invitrogen, St. Louis, MO) following the manufacturer’s instructions. RNA was treated with DNAse I (Fermentas, Waltham, MA) and first-strand cDNA synthesis was carried out using High-Capacity cDNA Reverse transcription kit (Applied Biosystems, St. Louis, MO). The amplification efficiency of the experimental set for each gene was tested with serial dilutions of cDNA and was only used if the resultant efficiency was ≥ 90%. Each PCR reaction (15 μL volume) contained diluted cDNA, Power SYBR Green PCR Master Mix (Applied Biosystems, St. Louis, MO) and 300 nM of forward and reverse primers. Quantitative PCR was performed in a StepOnePlus Real Time PCR System (Applied Biosystems, St. Louis, MO) using Applied Biosystems recommended qPCR conditions (20 seconds at 95 ^o^ C followed by 40 cycles of 95 ^o^ C for 1 second and 20 seconds at 60 ^o^ C followed by a melting curve to assure a single product was amplified). The comparative ΔΔCt method was used to evaluate changes in gene expression levels and all standard errors were calculated based on ΔCt as described in Applied Biosystems User Bulletin #2 (http://www3.appliedbiosystems.com/cms/groups/mcb_support/documents/generaldocuments/cms_040980.pdf). The *A. aegypti* ribosomal protein 49 gene, RP-49, was used as endogenous control (accession number AAT45939), based on previous data^48^. Each figure represents at least five biological replicates with three technical replicates for each sample. Oligonucleotides used in this study were listed in Supplementary table S1.

### Statistical analysis

All analyses were performed with GraphPad Prism statistical software package (Prism 5.01, GraphPad Software, Inc., San Diego, CA). Asterisks indicate significant differences (*p < 0.05; **p < 0.01; ***p < 0.001) and the type of test used in each analysis is described in its respective figure legend.

## Additional Information

**How to cite this article**: Barletta, A. B. F. *et al.* Emerging role of lipid droplets in *Aedes aegypti* immune response against bacteria and Dengue virus. *Sci. Rep.* **6**, 19928; doi: 10.1038/srep19928 (2016).

## Supplementary Material

Supplementary Information

Supplementary dataset S1
