# Supplementary material for: Emerging role of lipid droplets in Aedes aegypti immune response against bacteria and Dengue virus
Source: Sci Rep. 2016 Feb 18;6:19928. doi: 10.1038/srep19928 (PMC4757862; doi:10.1038/srep19928)
Supplement: Supplementary Information [file srep19928-s1.pdf]

Supplementary Information

Emerging role of lipid droplets in *Aedes aegypti* immune response against bacteria and Dengue virus

Ana Beatriz Barletta Ferreira[1,2], Liliane Rosa Alves[3], Maria Clara L. Nascimento Silva[1], Shuzhen Sim[4], George Dimopoulos[4], Sally Liechocki[5], Clarissa M. Maya-Monteiro[5], and Marcos H. Ferreira Sorgine[1,2*].

Figure S1

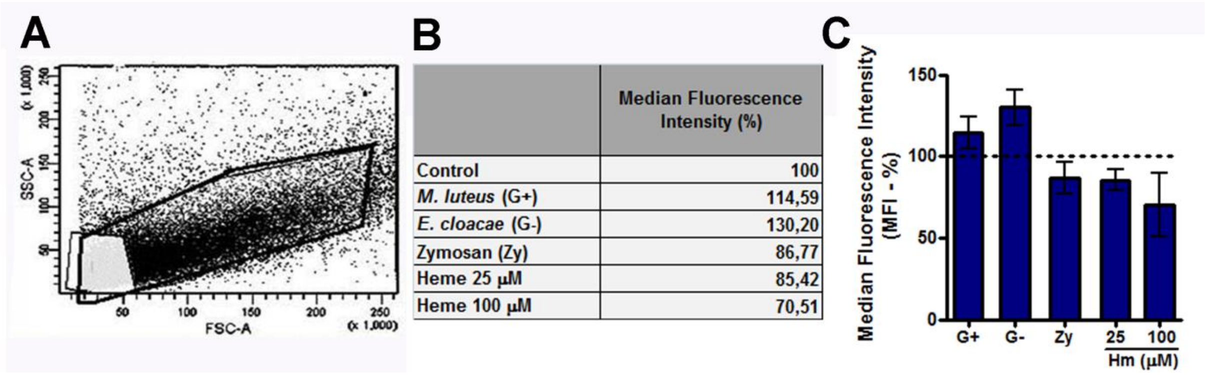

	Median Fluorescence Intensity (%)
Control	100
M. luteus (G+)	114,59
E. cloacae (G-)	130,20
Zymosan (Zy)	86,77
Heme 25 µM	85,42
Heme 100 µM	70,51

Figure S1: LDs can be differentially modulated by different stimuli.
Aag2 cells were incubated for 24 hours with heat-killed gram positive (G+, *Micrococcus luteus*) or gram negative (G-, *Enterobacter cloacae*) bacteria, zymosan or heme. After this period, cell LDs were stained with BODIPY 493/503 and the fluorescence intensity was measured by flow cytometry using a BD FACS Canto II flow cytometer (Beckton Dickinson, EUA). Data was analyzed using Infinicyt software (Cytognos, Spain). (A) Scatter plot distribution of Aag2 cells based on its size and granulosity - FSC – Forward scatter, SSC Side scatter. (B) Table showing the median fluorescence intensity of each group. (C) Graph representing the data shown in B.

Figure S2

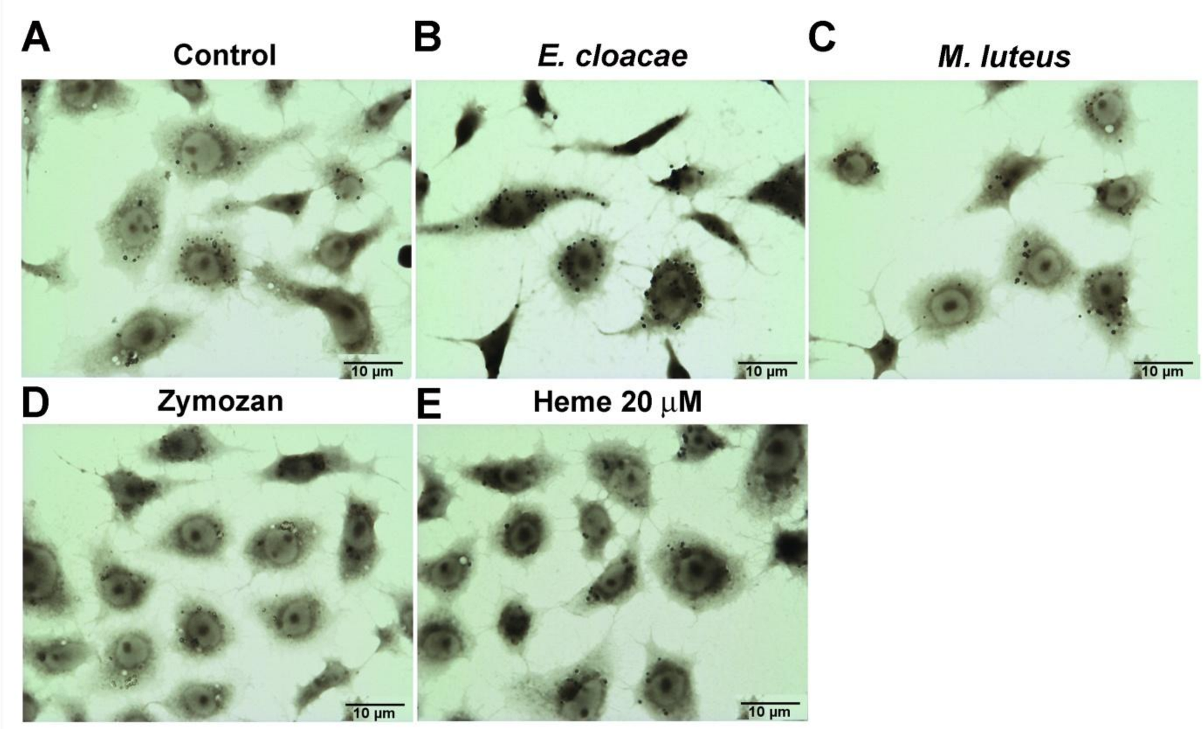

Figure S2: LDs can be differentially modulated by different stimuli.
Aag2 cells were were incubated for 24 hours with heat-killed gram positive (*M.luteus*) or gram negative (*E. cloacae*) bacteria, zymosan or heme. After the incubations cells were stained with Osmium Tetroxide and the number of LDs was visualized and counted under a light microscope. Lipid droplets can be seen as little black dots in the cytoplasm of the cell. (A) Control cells. (B) Gram negative bacteria (*E. cloacae*) (C) Gram positive bacteria (*M. luteus*). (D) Zymosan. (E) Heme.

Figure S3

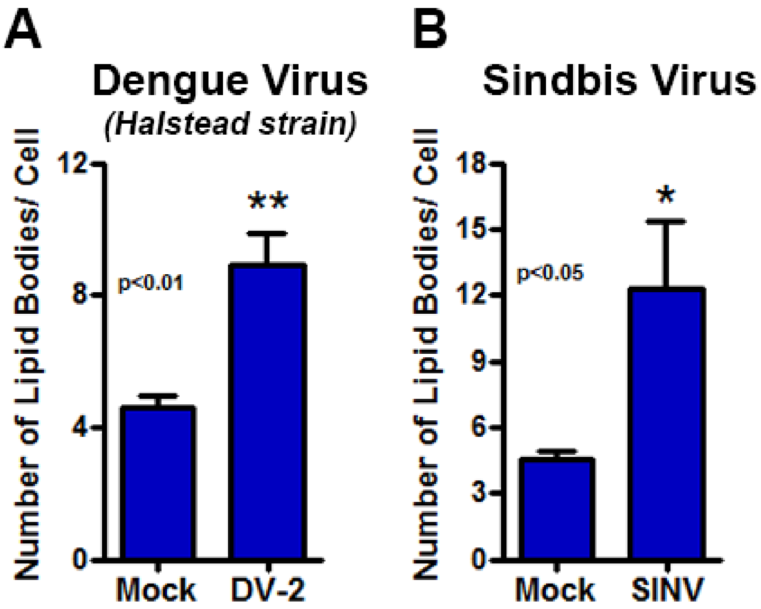

Figure S3: Viral infection leads to an increase in the number of cytoplasmic LDs in Aag2 cells.
Cells were mock infected or infected with either (A) Dengue 2 virus (Halstead strain) for 7 days or with (B) Sindbis virus for 4 days and stained with osmium tetroxide for estimation of the number of cytoplasmic LDs. 50 cells were randomically counted in each condition. For statistical analyzes, unpaired t-test was used.

Figure S4

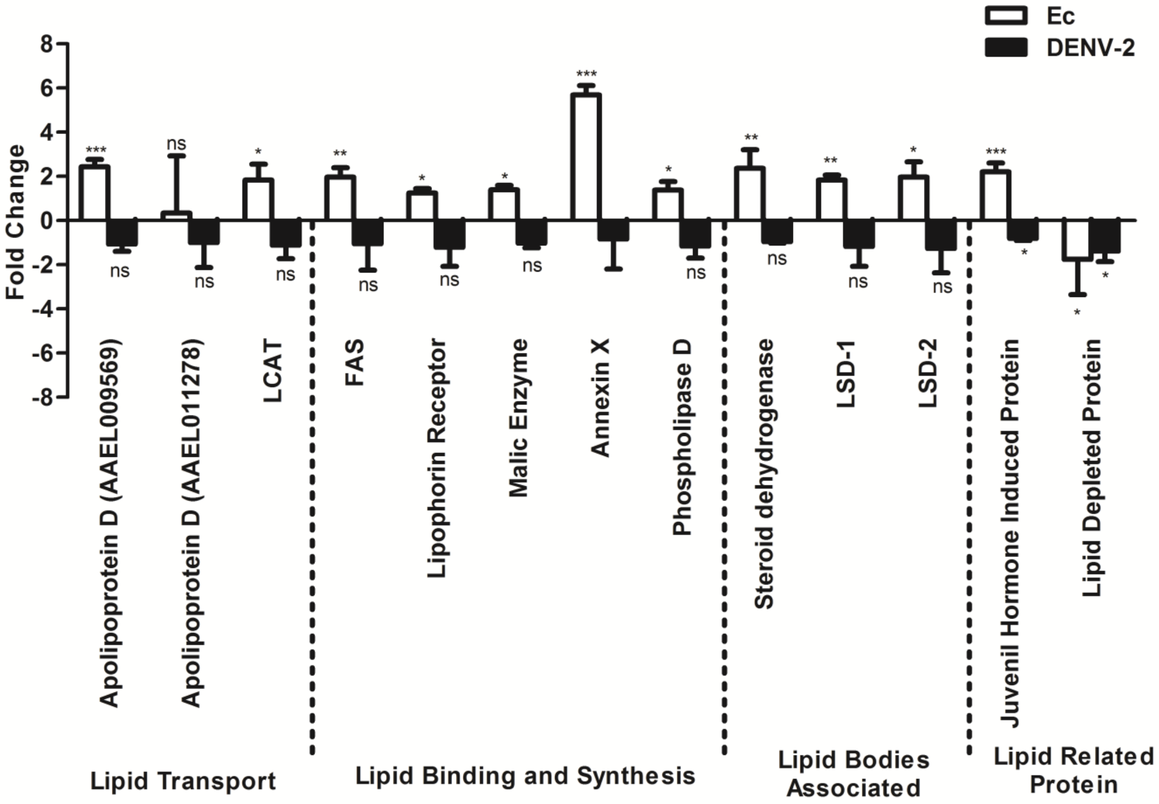

Figure S4: Validation of the microarray shown in figure 4.
Thirteen of the genes identified on the microarray assay shown in Figure 4 were chosen for validation of the assay. Aag2 cells were exposed to either heat-killed *Enterobacter cloacae* (Gram negative, Ec) for 6 hours or to Dengue virus for 4 days. After these periods total RNA was extracted from the cells and the expression of 13 genes was analyzed by qPCR. Statistical analyses were conducted using unpaired t-test in order to compare the differences between biological replicates.

Figure S5

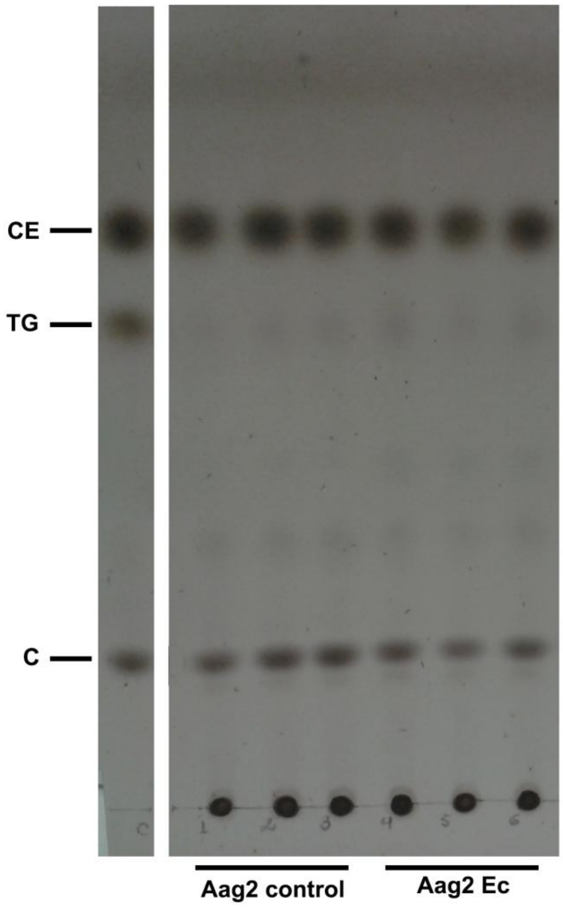

Figure S5: Identification of neutral lipid species by Thin Layer Chromatography (TLC).
Representative TLC of neutral lipids content of control and heat killed bacteria stimulated (24 hours) Aag2 cells. C –Cholesterol; TG – Triacylglycerol; CE – Cholesteryl ester. Three independent experiments for each condition were fractionated on the TLC as indicated in the figure.

Table S1

Table S1: List of primers used in this study.

Gene	Primer Foward	Primer Reverse	Accession Number
**RP49 qPCR**	GCTATGACAAGCTTGCCCCCA	TCATCAGCACCTCCAGCT	AAEL003396
**Lipid depleted protein qPCR**	AACCTTGCGGACAACAAATC	CTCCTTCAGACGCTTTTTGG	AAEL009309
**Lipophorin receptor qPCR**	GCGTTGATCCGTCTCTTAGC	ATTTGGCAGGAGTGTTCCAC	AAEL012251
**Malic Enzyme qPCR**	AACACGGTTCTGTTCCAAGG	CCTTCGGACGATCCTTAACA	AAEL005790
**Fatty Acid Synthase (FAS) qPCR**	CGGTTTGGTGTTGGAGAACT	GAATCGGACACGTTCTTGGT	AAEL001194
**Phosphatidylcholine-sterol acyltransfer**	GAGCGTACTGGAACAGCATCA	CGCTGCCAAGACATTCTACA	AAEL013170
**Apolipoprotein D qPCR**	CCCTTTGACCTCAGCATCAT	ATTCACGGCATACTCGAACC	AAEL013574
**Apolipoprotein D qPCR**	TTCTCCTGTTCGGGACAATC	ATCCTCCAGCTCGACATCAT	AAEL009569
**Steroid dehydrogenase qPCR**	GATGGAATTGGCAAAGCCTA	GTTTCGTAGATYTCCGGTCC	AAEL009634
**Juvenile hormone-inducible protein qP**	ATCTTCTGCACGGTCGAAGT	TGCCTCTTGATGTCTGCTTG	AAEL014438
**Annexin x qPCR**	TGATCCAGATCTTGCTGTGC	GATGGTACGTCCGGTCAGTT	AAEL005417
**Phospholipase D qPCR**	GTCGGTGGTGCTAGTGGAAT	CAGCAATCAACAGCTTGGAA	AAEL000264
**LSD-1 (Perilipin -1) qPCR**	CCGATCATCAAGGAACAACC	CAGTAGACGCTCGGCCAGT	AAEL009231
**LSD-2 (Perilipin -2) qPCR**	TGGAAGAAAGCCAACGAAG	AGGGCTGGATCTTCCTTGA	AAEL006820
**dsRNA Cactus**	**TAATACGACTCACTATAGGG** CGAGTCAACAGAACCCGAGCAG	**TAATACGACTCACTATAGGG** TGGCCCGTCAGCACCGAAAG	AAEL000709
**dsRNA Caspar**	**TAATACGACTCACTATAGGG** GGAAGCAGATCGAGCCAAGCAG	**TAATACGACTCACTATAGGG** GCATTGAGCCGCCTGGTGTC	AAEL003579
**Cactus qPCR**	AGACAGCCGCACCTTCGATTCC	CGCTTCGGTAGCCTCGTGGATC	AAEL000709
**Caspar qPCR**	GAATCCGAGCGAGCCGATGC	CGTAGTCCAGCGTTGTGAGGTC	AAEL003579

Dataset S1: Expression changes of Aag2 genes modulated after bacterial or viral infection.
